# Synthesis, Characterization and Photocatalytic Activity of New Photocatalyst ZnBiSbO_4_ under Visible Light Irradiation

**DOI:** 10.3390/ijms15069459

**Published:** 2014-05-28

**Authors:** Jingfei Luan, Mengjing Chen, Wenhua Hu

**Affiliations:** State Key Laboratory of Pollution Control and Resource Reuse, School of the Environment, Nanjing University, Nanjing 210093, China; E-Mails: custcmj@gmail.com (M.C.); wenhuahu2000@163.com (W.H.)

**Keywords:** ZnBiSbO_4_, indigo carmine, photocatalytic degradation, visible light irradiation, photodegradation pathway

## Abstract

In this paper, ZnBiSbO_4_ was synthesized by a solid-state reaction method for the first time. The structural and photocatalytic properties of ZnBiSbO_4_ had been characterized by X-ray diffraction, scanning electron microscopy, X-ray photoelectron spectroscopy, Fourier-transform infrared spectroscopy, transmission electron microscope and UV-visible spectrometer. ZnBiSbO_4_ crystallized with a pyrochlore-type structure and a tetragonal crystal system. The band gap of ZnBiSbO_4_ was estimated to be 2.49 eV. The photocatalytic degradation of indigo carmine was realized under visible light irradiation with ZnBiSbO_4_ as a catalyst compared with nitrogen-doped TiO_2_ (N-TiO_2_) and CdBiYO_4_. The results showed that ZnBiSbO_4_ owned higher photocatalytic activity compared with N-TiO_2_ or CdBiYO_4_ for the photocatalytic degradation of indigo carmine under visible light irradiation. The reduction of the total organic carbon, the formation of inorganic products, SO_4_^2−^ and NO_3_^−^, and the evolution of CO_2_ revealed the continuous mineralization of indigo carmine during the photocatalytic process. One possible photocatalytic degradation pathway of indigo carmine was obtained. The phytotoxicity of the photocatalytic-treated indigo carmine (IC) wastewater was detected by examining its effect on seed germination and growth.

## 1. Introduction

Dye effluents from textile industries and photographic industries are becoming a serious environmental problem because of their toxicity, unacceptable color, high chemical oxygen demand content and biological degradation [1]. The presence of dye in water is not only aesthetically displeasing, but also affects water transparency, which results in the reduction of sunlight penetration, gas solubility and the photosynthetic reaction [2]. In order to solve this problem, many scientists look forward to degrading harmful dye effluents from contaminated water with photocatalytic techniques before appropriate disposal, and these scientists have been contributing their various efforts to this career for more than 40 years [3,4,5]. Nowadays, photocatalytic degradation processes have been widely applied as techniques for the destruction of organic pollutants in wastewater and effluents, especially the degradation of dyes [6].

The choice of the wavelength of incident light is important for a photocatalytic degradation system, because light was the source of energy. Previous studies have shown that such semiconductor compounds could degrade most kinds of persistent organic pollutants, such as dyes, pesticide, detergents and volatile organic compounds, under UV-light irradiation [7,8,9,10], which has a higher energy than visible light. As is well known, ultraviolet light occupies only 4% of sunlight; on the other hand, visible light accounts for a share of about 43%. Thus, it seems more practical and favorable to use visible light rather than ultraviolet light for the degradation process. Therefore, it is necessary and urgent to make substantive efforts to develop new photocatalysts, which have a response to visible light and show a relatively high efficiency of degradation. In general, the majority of visible light-driven catalysts in previous research could mainly be classified into two types: one kind is called the TiO_2_-based catalyst, whose maximum absorption wavelength had been extended to visible light by ion doping [11,12,13,14,15,16,17,18,19,20] and cocatalyst recombination [21,22,23,24,25,26,27,28,29,30,31]; the other one is a multiple complex oxide, such as BiVO_4_, Bi_12_TiO_20_, K_6_Nb_10.8_O_30_, *etc.* [32,33,34,35,36,37,38]. Recently, a spinel type oxide with a formula of AB_2_O_4_ had been found to perform excellent for degrading dyes that existed in wastewater under visible light irradiation. For example, MIn_2_O_4_ (M = Ca, Sr, Ba) [11,39,40], NiCo_2_O_4_ [11] and ZnFe_2_O_4_/MWCNTs [41] were synthesized for methylene blue degradation under visible light irradiation. Additionally, ZnFe_2_O_4_ [42] was also reported to perform outstandingly for methyl orange degradation under visible light irradiation.

In this paper, newly synthesized semiconductor catalyst ZnBiSbO_4_ which belongs to the AB_2_O_4_ compound family is presented. We choose indigo carmine (IC) as the model pollutant to evaluate the degradation activity of ZnBiSbO_4_ under visible light irradiation, because indigo carmine is widely applied and difficult for biodegradation. Additionally, the structural and photocatalytic properties of ZnBiSbO_4_ have been investigated in detail. For comparison, we select N-TiO_2_, one kind of traditional photocatalyst, as a catalyst for IC under visible light irradiation. Besides, comparative experiments have been conducted by using CdBiYO_4_ [43], one representative catalyst of the AB_2_O_4_-type compounds, which has been published in our former work, to demonstrate further the superiority of the degradation activity of ZnBiSbO_4_.

## 2. Results and Discussion

### 2.1. Crystal Structure and Optical Properties

**Figure 1:**
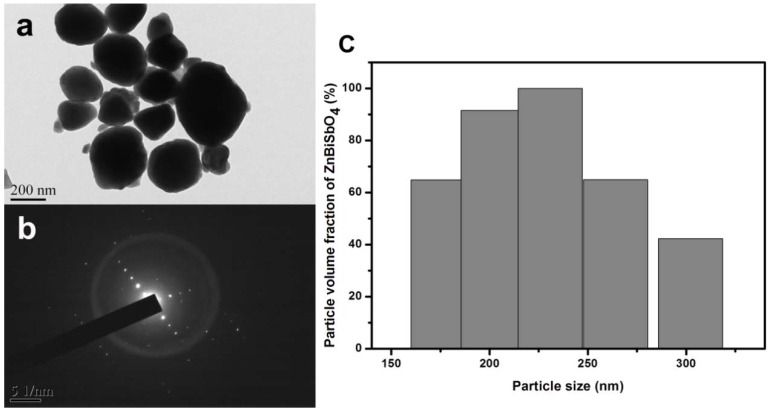
(**a**) transmission electron microscopy (TEM) image of ZnBiSbO_4_; and (**b**) The selected area electron diffraction pattern of ZnBiSbO_4_; and (**c**) histogram of the size distribution of ZnBiSbO_4_ calcinated at 800 °C for 35 h.

The transmission electron microscopy (TEM) image of the prepared catalyst, ZnBiSbO_4_, is shown in Figure 1. It could be observed clearly from Figure 1a,b that the particles of ZnBiSbO_4_ had a nanostructure and irregular shapes. Additionally, we could also acknowledge that the particles of ZnBiSbO_4_ crystallized well, and the average particle size was about 220 nm in diameter. Figure 1b also shows the selected area electron diffraction pattern of ZnBiSbO_4_. It could be seen from Figure 1b that ZnBiSbO_4_ crystallized with a pyrochlore-type structure, a tetragonal crystal system and a space group I41/A, and the lattice parameters for ZnBiSbO_4_ were proven to be *a* = *b* = 12.040078 Å and *c* = 10.731416 Å. According to the calculation results from Figure 1b, the (*h k l*) value for the main peaks of ZnBiSbO_4_ could be found and indexed. The histogram of the size distribution of ZnBiSbO_4_ was also demonstrated in Figure 1c, and we could find that the particle size of the majority of ZnBiSbO_4_ was from 170 to 270 nm. Brunauer–Emmett–Teller (BET) measurements were also detected, and the specific surface area of ZnBiSbO_4_ was 2.87 m^2^·g^−1^.

**Figure 2:**
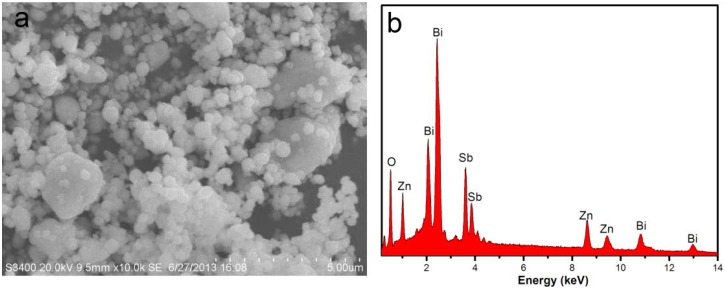
(**a**) scanning electron microscopy (SEM) spectrum of ZnBiSbO_4_; (**b**) energy-dispersive spectrometry (EDS) spectrum of ZnBiSbO_4_ calcinated at 800 °C for 35 h.

Figure 2 presents the scanning electron microscopy-energy-dispersive spectrometry (SEM-EDS) spectrum of ZnBiSbO_4_, indicating the presence of zinc, bismuth, antimony and oxygen element. In order to avoid the influence of inhomogeneity phenomenon on the selected surface, ten different specimen areas selection of ZnBiSbO_4_ were conducted in an EDS test. The mean of the results of the above EDS spectra taken from prepared ZnBiSbO_4_ indicated that the stoichiometric ratio of zinc, bismuth, antimony and oxygen was estimated to be 14.83:14.55:13.56:57.06, namely 1.05:1.03:0.96:4.04. 

**Figure 3:**
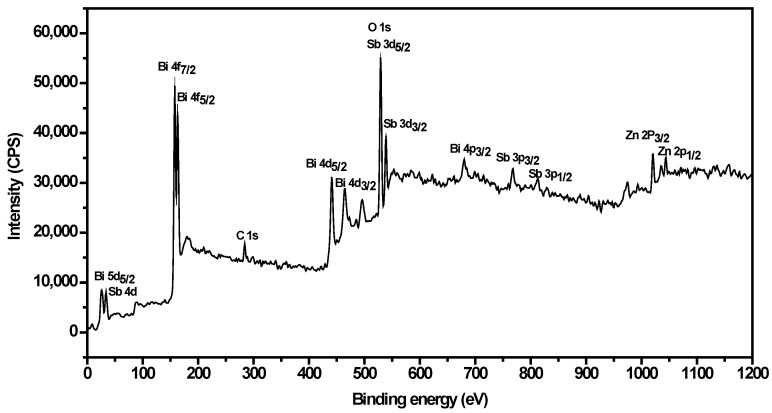
The X-ray photoelectron spectroscopy (XPS) full spectrum of ZnBiSbO_4_ calcinated at 800 °C for 35 h.

**Figure 4:**
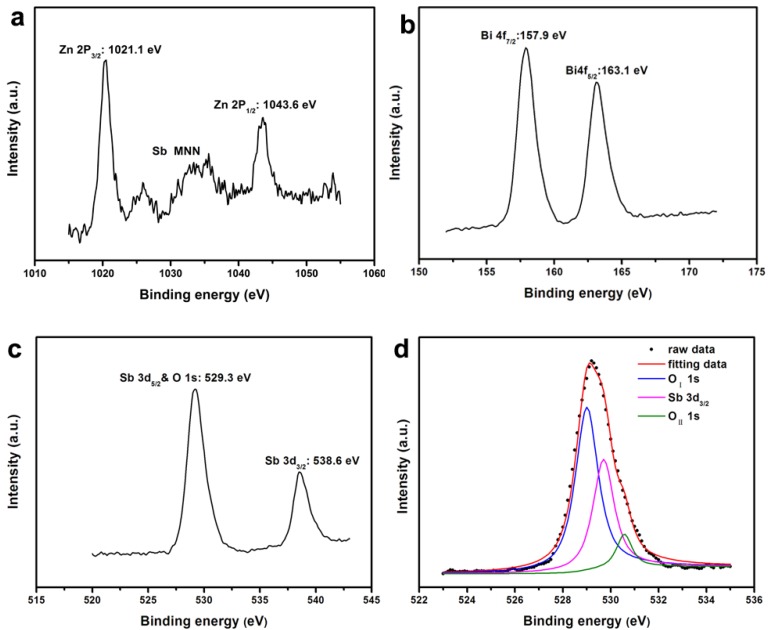
(**a**) The XPS spectrum of Zn 2p; (**b**) the XPS spectrum of Bi 4f; (**c**) the XPS spectrum of Sb 3d; and (**d**) peak curves of Sb 3d_5/2_ and O 1s for the XPS spectrum of ZnBiSbO_4_ (O_I_, lattice oxygen; O_II_, chemisorbed oxygen).

In order to get a better understanding of the chemical state of all elements on the catalyst surface, the X-ray photoelectron spectroscopy (XPS) full spectrum of ZnBiSbO_4_ was measured and is displayed in Figure 3. The atom XPS spectra of Zn, Bi and Sb are shown in Figure 4a–c, respectively. As to the O atom XPS spectrum, we could observe that the O 1s peak was asymmetric and should be overlaid by the Sb 3d_5/2_ peak and the O 1s peak. Besides lattice oxygen (O_I_) in multiple oxide catalyst, the existence of chemisorbed oxygen (O_II_) on the surface of the catalyst should also be taken into consideration [44]. Thus, the fitted curves of Sb 3d_5/2_ and O 1s for XPS spectra are given in Figure 4d, and the fitting results are reported in Table 1. The various elemental peaks, which correspond to the specific binding energies of ZnBiSbO_4_, are given in Table 2. The area ratio of O_I_/O_T_ was 87.36% (O_T_ contained O_I_ and O_II_), meaning that the atom ratio of O_I_/O_T_ is 87.36% on the catalyst surface. The results further suggested that the oxidation state of Zn, Bi, Sb and O ions from ZnBiSbO_4_ were +2, +3, +3 and −2, respectively. After calculation with the reported method [45], the average atomic ratio of Zn:Bi:Sb:O for ZnBiSbO_4_ was 1.00:0.98:1.02:4.12 based on our XPS results. The slight difference between the results that were found in XPS and EDS measurements could be ascribed to the inhomogeneity of different selected areas of ZnBiSbO_4_ during the testing. Therefore, it could be deduced that the obtained material was of high purity under our preparation conditions.

**Table 1:** XPS data corresponding to Figure 4d.

Element	Sb 3d_5/2_	O 1s	
		O_I_	O_II_
Binding energy (eV)	529.7	529.0	530.6

**Table 2:** Binding energies for key elements of ZnBiSbO_4_.

Element	Zn 2p_3/2_	Bi 4f_7/2_	Sb 3d_5/2_	O 1s
Binding energy (eV)	1021.1	157.9	529.7	529.0

**Figure 5:**
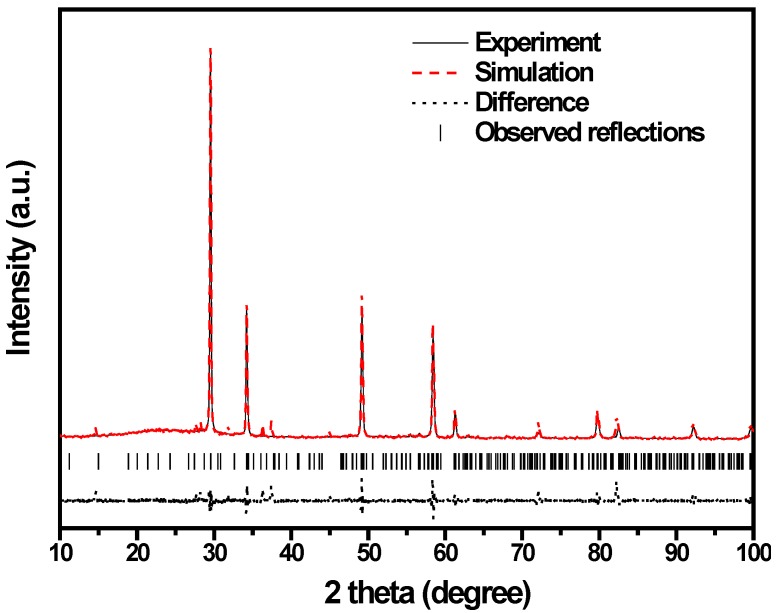
X-ray diffraction (XRD) pattern and Rietveld refinements of ZnBiSbO_4_.

**Figure 6:**
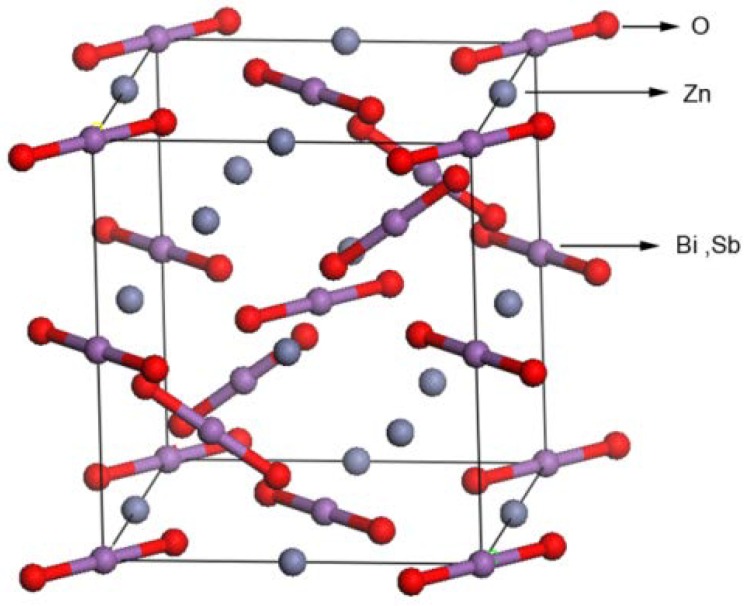
The structural model of ZnBiSbO_4_ simulated by Materials Studio software corresponding to the XRD pattern shown in Figure 5.

Figure 5 shows the powder X-ray diffraction pattern of ZnBiSbO_4_ with the full-profile structure refinements of the collected data, which were obtained by the RIETAN™ [46] program based on the Rietveld analysis. It could be seen from Figure 5 that ZnBiSbO_4_ turned out to be a single phase. Additionally, the results of the final refinements for ZnBiSbO_4_ indicated a good agreement between the observed intensities and calculated intensities for the pyrochlore-type structure, a tetragonal crystal system and a space group, I41/A (O atoms were included in the model). The lattice parameters for ZnBiSbO_4_ were *a* = *b* = 12.040078 Å and *c* = 10.731416 Å. All the diffraction peaks for ZnBiSbO_4_ could be successfully indexed according to the lattice constant and above space group. The atomic coordinates and structural parameters of ZnBiSbO_4_ are listed in Table 3. According to above results, the structural model of ZnBiSbO_4_ which is simulated by Materials Studio software is demonstrated in Figure 6.

**Table 3:** Structural parameters of ZnBiSbO_4_ calcinated at 800 °C for 35 h.

Atom	*x*	*y*	*z*	Occupation factor
Zn	0	0	0.5	1
Bi	0	0	0	1
Sb	0	0	0	1
O	0.76731	0.14013	0.08188	1

**Figure 7:**
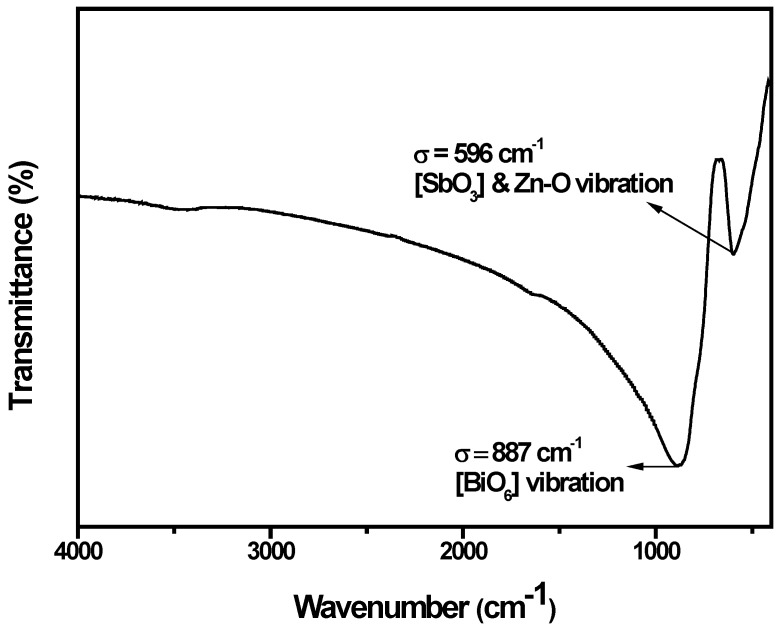
Fourier transform infrared (FTIR) spectrum of ZnBiSbO_4_ calcinated at 800 °C for 35 h.

Fourier transform infrared (FTIR) spectrum analysis of ZnBiSbO_4_ particles is investigated in this study, as shown in Figure 7. From this picture, we could find that the absorption bands of ZnBiSbO_4_ prepared by a solid-state reaction method at 800 °C are at 596 and 887 cm^−1^. The strong band near 887 cm^−1^ should be attributed to the Bi–O vibration in the distorted [BiO_6_] unit [47]. The band situated at 596 cm^−1^ was overlaid by the symmetric bending and stretching of the [SbO_3_] unit [48] and Zn–O stretching [49].

**Figure 8:**
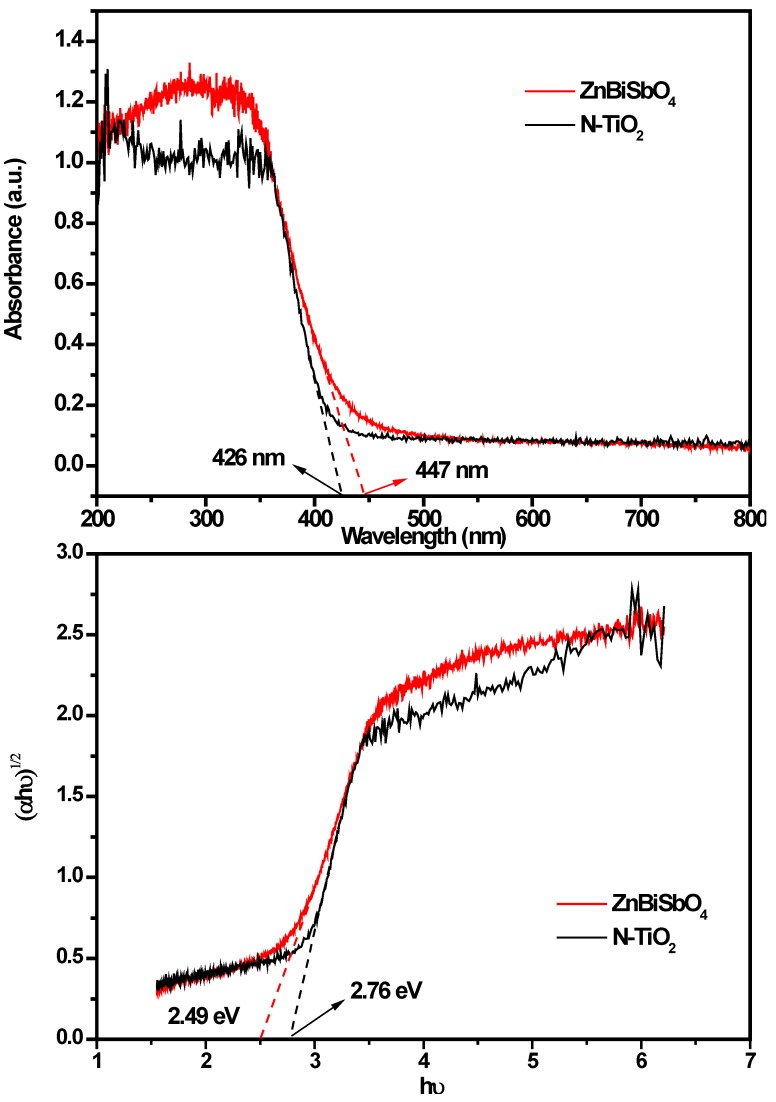
The absorption spectrum, plot of (α*h*ν)^1/2^ *vs.* *h*ν for ZnBiSbO_4_ calcinated at 800 °C for 35 h and N-TiO_2_.

The absorption spectrum of ZnBiSbO_4_ is presented in Figure 8. For a crystalline semiconductor, the optical absorption near the band edge following Equation (1) [50]:

α*hν* = *A* × (*hν* − *E*_g_)*^n^* (1)

*E*_g_ = 1240/λ (2)

Here, *A*, α, *E*_g_, ν and λ were the proportional constant, absorption coefficient, band gap, light frequency and absorption edge, respectively. In this equation, n determined the character of the transition in a semiconductor. *E*_g_ and *n* could be calculated by the following steps [51]: (i) plotting ln (α*h*ν) *vs.* ln (*h*ν − *E*_g_) by assuming an approximate value of *E*_g_, which can be calculated by Equation (2); (ii) deducing the value of *n*; and (iii) refining the value of *E*_g_. From Figure 8, we could find that the absorption edge of ZnBiSbO_4_ was about 447 nm, meaning that the estimated *E*_g_ of ZnBiSbO_4_ was 2.78 eV. Then, plot ln (α*h*ν) *vs.* ln (*h*ν − *E*_g_), where we could find the slope of the line part, was about 1.65. Therefore, the *n* of ZnBiSbO_4_ was two. After plotting (α*hν*)^1/2^ *vs.* *h*ν and extrapolating the plot to (α*h*ν)^1/2^ = 0, the accurate value of *E*_g_ of ZnBiSbO_4_ was calculated as 2.49 eV. Applying the same calculation process to N-TiO_2_, we found that for N-TiO_2_: *n* = 2 and *E*_g_ = 2.76 eV. The above results indicated that the optical transition for ZnBiSbO_4_ or N-TiO_2_ was indirectly allowed, and ZnBiSbO_4_ possessed a narrow band gap compared with N-TiO_2_. The results in our past work showed that the optical transition for CdBiYO_4_ was directly allowed and that the band gap of CdBiYO_4_ was 2.41 eV [43].

### 2.2. Photocatalytic Properties

The progress of photocatalysis using the semiconductor compound could be described briefly as follows [52,53]. Firstly, the semiconductor compound absorbed photons, resulting in the generation of electron-hole pairs within the semiconductor compound particles and, subsequently, the diffusion of the charge carriers to the surface of the semiconductor compound particle would be followed; at the same time, the active sites of the surface of the semiconductor compound particles had been adsorbing a lot of pollutants particles; finally, the decomposition of pollutants would be performed by charge carriers.

**Figure 9:**
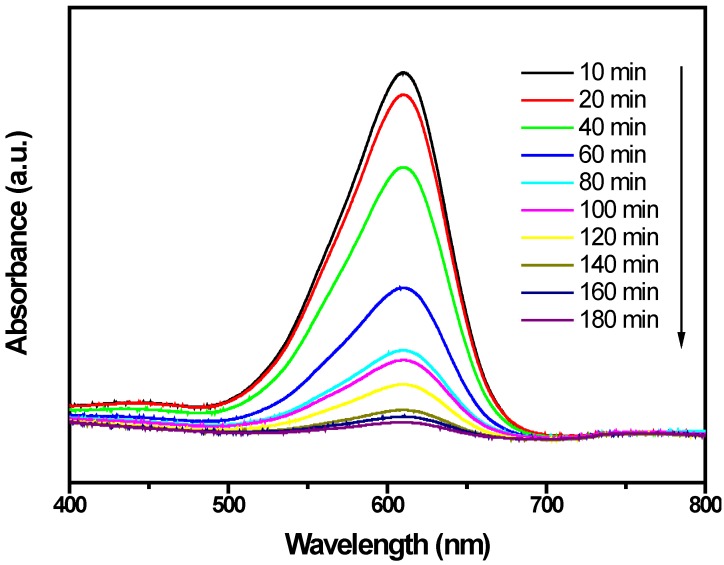
Spectral changes of aqueous solutions of indigo carmine (IC) due to visible light irradiation with the presence of ZnBiSbO_4_ calcinated at 800 °C for 35 h.

**Figure 10:**
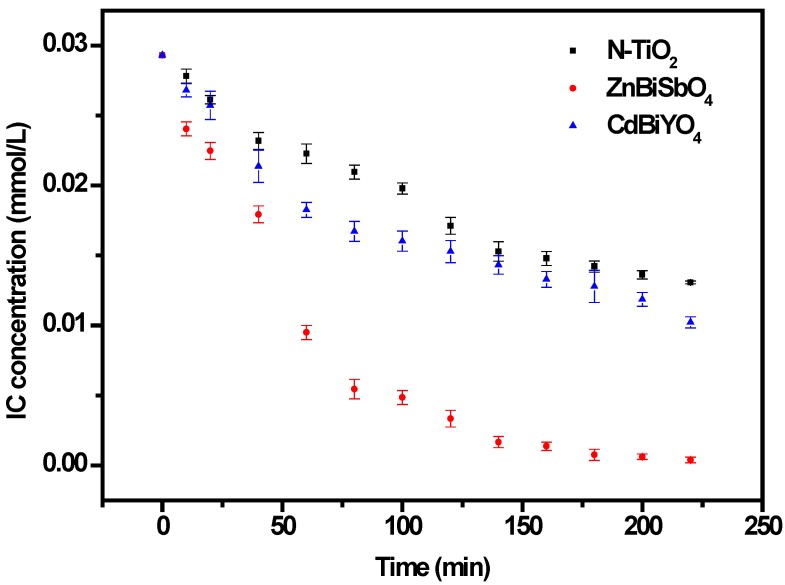
Photocatalytic degradation of IC under visible light irradiation with the presence of ZnBiSbO_4_, CdBiYO_4_ and N-TiO_2_ as photocatalysts, respectively. The results are means of triplicates, and error bars represent ±1 SD.

Figure 9 presents the changes in the UV-Vis spectra of IC under visible light irradiation (λ > 400 nm) with the presence of ZnBiSbO_4_. The above measurements were performed under oxygen-saturation conditions ([O_2_]_sat_ = 1.02 × 10^−3^ M). It could be clearly noticed from Figure 9 that the typical IC peaks were at 609.5 nm. An obvious color change from deep red into a colorless solution could be observed within 230 min. For further comparison, Figure 10 depicts the concentration changes of IC with ZnBiSbO_4_, CdBiYO_4_ and N-TiO_2_ as photocatalysts under visible light irradiation, respectively. It could be seen from Figure 10 that the photonic efficiency (λ = 420 nm) was estimated to be 0.0460%, 0.0258% and 0.0304% with ZnBiSbO_4_, N-TiO_2_ and CdBiYO_4_, respectively. When ZnBiSbO_4_ or CdBiYO_4_ or N-TiO_2_ was utilized as a catalyst, the photodegradation conversion rate of IC was 98.66% or 65.12% or 55.39% after visible light irradiation for 220 min. The results showed that the photodegradation rate of IC and the photonic efficiency with ZnBiSbO_4_ as a catalyst were both higher than those with N-TiO_2_ or CdBiYO_4_ as a catalyst. The above results showed that complete removal of indigo carmine was observed after visible light irradiation for 230 min with ZnBiSbO_4_ as a catalyst. Besides, based on the absorbance changes of IC with irradiation time, the kinetics of IC degradation under visible light irradiation was figured out. The above results demonstrated that the photocatalytic kinetics of IC degradation with ZnBiSbO_4_, CdBiYO_4_ and N-TiO_2_ as photocatalysts followed a first order nature. The first-order rate constants for IC degradation were estimated to be 0.01945, 0.00405 and 0.00502 min^−1^ with ZnBiSbO_4_, N-TiO_2_ and CdBiYO_4_ as catalysts, respectively. This fact indicated that ZnBiSbO_4_ was more efficient than N-TiO_2_ or CdBiYO_4_ for the photocatalytic degradation of IC under visible light irradiation.

**Figure 11:**
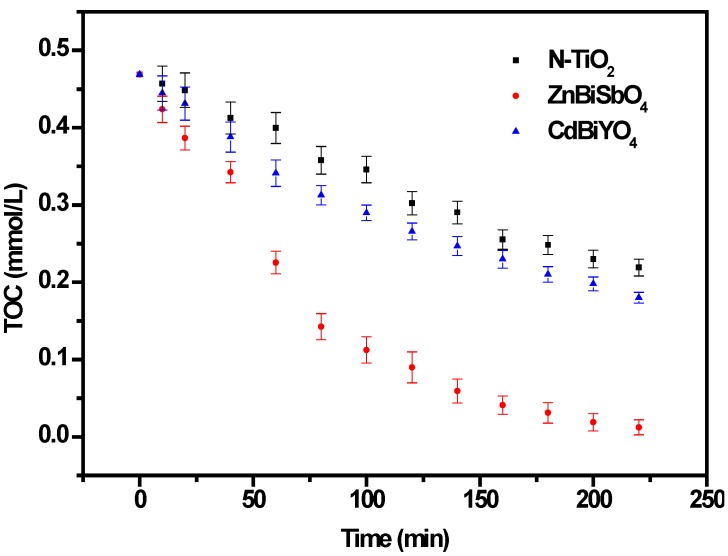
Total organic carbon (TOC) plots during the photocatalytic degradation of IC under visible light irradiation with ZnBiSbO_4_, CdBiYO_4_ and N-TiO_2_ as photocatalysts, respectively. The results are means of triplicates, and error bars represent ±1 SD.

Figure 11 shows the change of total organic carbon (TOC) during the photocatalytic degradation of IC with ZnBiSbO_4_, CdBiYO_4_ or N-TiO_2_ as a catalyst under visible light irradiation. The TOC measurements revealed the disappearance of organic carbon when the IC solution, which contained ZnBiSbO_4_ or CdBiYO_4_ or N-TiO_2_, was exposed under visible light irradiation. The results showed that 97.37% or 61.59% or 53.26% of a TOC decrease was obtained after visible light irradiation for 220 min when ZnBiSbO_4_ or CdBiYO_4_ or N-TiO_2_ was utilized as the photocatalyst. Consequently, it could be seen from Figure 11 that the entire mineralization of indigo carmine was realized after visible light irradiation for 270 min with ZnBiSbO_4_ as a catalyst, because of the 100% TOC removal. The apparent first order rate constant, *k*, was estimated to be 0.01536, 0.00449 and 0.00350 min^−1^ with ZnBiSbO_4_, CdBiYO_4_ or N-TiO_2_ as the photocatalyst, respectively.

**Figure 12:**
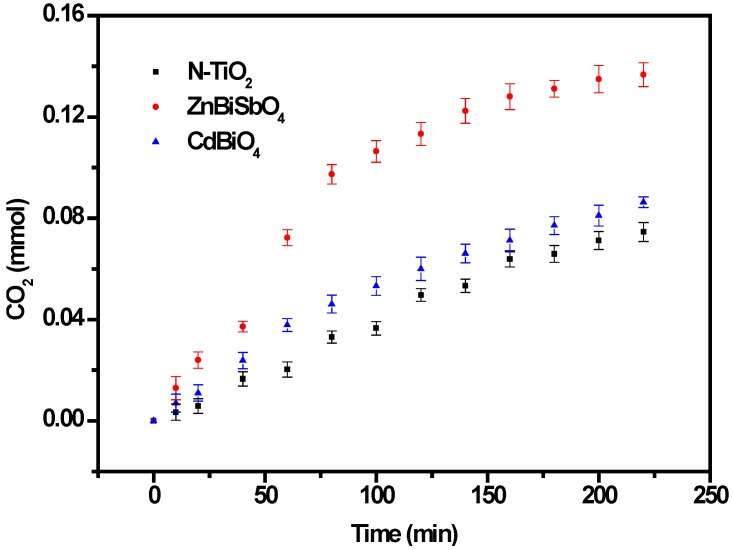
CO_2_ production during the photocatalytic degradation of IC with ZnBiSbO_4_, CdBiYO_4_ and N-TiO_2_ as photocatalysts, respectively, under visible light irradiation. The results are means of triplicates, and error bars represent ±1 SD.

During the progress of IC degradation, IC was converted into smaller organic species and was ultimately mineralized to inorganic products, such as carbon dioxide and water. Figure 12 presents the CO_2_ yield during the photocatalytic degradation of IC with ZnBiSbO_4_ or CdBiYO_4_ or N-TiO_2_ as the photocatalyst under visible light irradiation. The amount of CO_2_ increased gradually with increasing reaction time when IC was photodegraded with ZnBiSbO_4_ or CdBiYO_4_ or N-TiO_2_ as the photocatalyst. The results showed that the production rate of CO_2_ from the ZnBiSbO_4_-IC system was higher than that from the CdBiYO_4_-IC system or N-TiO_2_-IC system with increasing reaction time. For example, the production amount of CO_2_ was 0.08103 or 0.07122 mmol with CdBiYO_4_ or N-TiO_2_ as the photocatalyst after a visible light irradiation of 200 min. However, the production amount of CO_2_ was 0.1349 mmol with ZnBiSbO_4_ as the photocatalyst after a visible light irradiation of 200 min.

**Figure 13:**
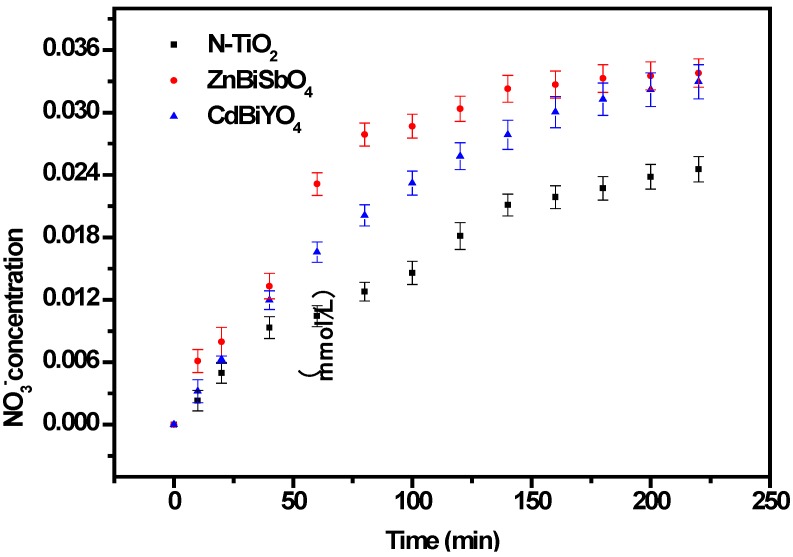
NO_3_^−^ production during the photocatalytic degradation of IC with ZnBiSbO_4_, CdBiYO_4_ and N-TiO_2_ as photocatalysts, respectively, under visible light irradiation. The results are means of triplicates, and error bars represent ±1 SD.

**Figure 14:**
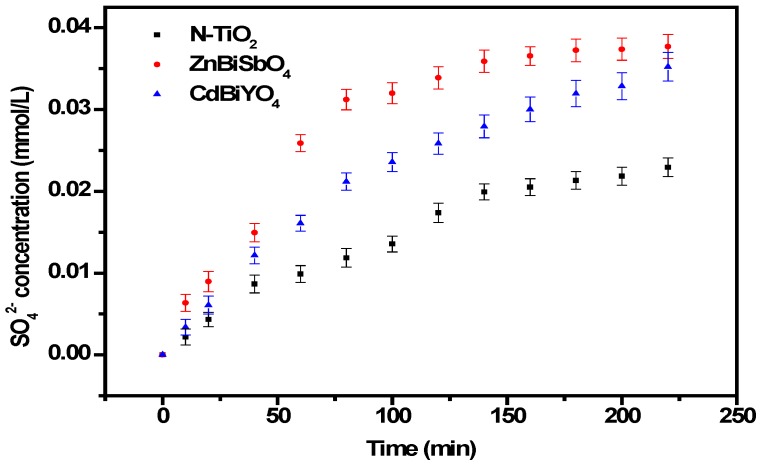
SO_4_^2−^ production during the photocatalytic degradation of IC with ZnBiSbO_4_, CdBiYO_4_ and N-TiO_2_ as photocatalysts, respectively, under visible light irradiation. The results are the means of triplicates, and error bars represent ±1 SD.

In order to explore the mechanism of the IC degradation with ZnBiSbO_4_ or CdBiYO_4_ or N-TiO_2_ as photocatalyst under visible light irradiation, we also tested the concentration of inorganic ions, NO_3_^−^ and SO_4_^2−^, which are shown in Figure 13 and Figure 14, which may be formed as the end products of nitrogen and sulfur atoms that existed in IC. From Figure 13 and Figure 14, we could be sure that both NO_3_^−^ and SO_4_^2−^ appeared during IC degradation with ZnBiSbO_4_ or CdBiYO_4_ or N-TiO_2_ as the photocatalyst. NO_3_^−^ and SO_4_^2−^ ions were generated more quickly and effectively with ZnBiSbO_4_ compared with N-TiO_2_ or CdBiYO_4_ as photocatalyst, which was in accord with the above analysis about the degradation progress of IC. According to the NO_3_^−^ concentration in Figure 13, we could calculate that 57.66% or 68.95% or 41.89% of sulfur from IC was converted into sulfate ions with ZnBiSbO_4_ or CdBiYO_4_ or N-TiO_2_ as the photocatalyst after visible light irradiation for 220 min. Meanwhile, it could be also concluded that 64.32% or 60.09% or 39.11% of sulfur from IC was converted into sulfate ions with ZnBiSbO_4_ or CdBiYO_4_ or N-TiO_2_ as photocatalyst after visible light irradiation for 220 min. It was noteworthy that the amount of SO_4_^2−^ and NO_3_^−^ that were released into the solution were sharply lower than the stoichiometry value of 100%. One possible reason could be a loss of sulfur-containing volatile compounds or SO_2_ for the S element and nitrogen-containing volatile compounds or NH_3_. The second possible reason was a partially irreversible adsorption of some SO_4_^2−^ and NO_3_^−^ on the surface of the photocatalyst, which had been observed by Lachheb *et al*. with titanium dioxide [54].

**Figure 15:**
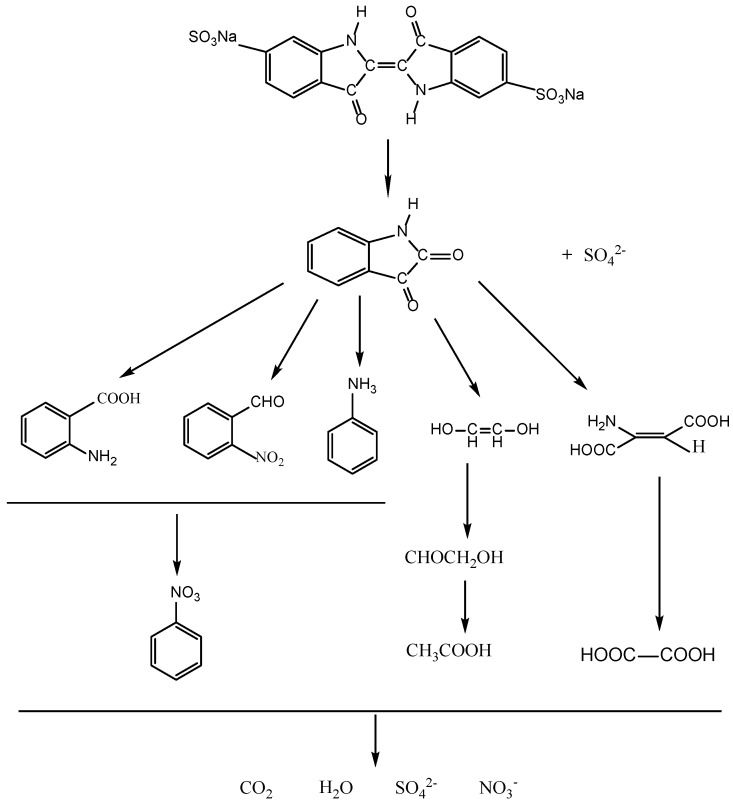
Suggested photocatalytic degradation pathway scheme for IC under visible light irradiation with the presence of ZnBiSbO_4_ prepared by a solid-state reaction method at 800 °C for 35 h.

In addition, the intermediates generated during the degradation process were detected by high-performance liquid chromatography (HPLC) and gas chromatography-mass spectrometry (GC-MS) and identified by comparison with commercial standard samples. The intermediates in our experiment were identified as follows: indoline-2,3-dione, anthranilic acid, *O*-nitrobenzyl dehyde, aniline, ethene-1,2-diol, amino-fumaric acid, oxalic acid, glycolal dehyde and acetic acid. The sulfur was first hydrolytically removed and subsequently was oxidized and transformed into SO_4_^2−^. At the same time, nitrogen atoms in the −3 oxidation state produced NH_4_^+^ cations that subsequently were oxidized into NO_3_^−^ ions. Based on the above results, we have deduced a degradation pathway of IC with ZnBiSbO_4_, as shown in Figure 15. This pathway was similar, but not identical, to the pathway proposed by Manon Vautier *et al*. [55]. According to Figure 15, the main degradation end-products of IC were CO_2_, NO_3_^−^ and SO_4_^2^. Thus, the mass balance of the C, N and S elements from IC (reaction time = 0 min) to end-products (reaction time = 220 min) during the degradation process of IC in the reactor had been studied. The original amount of C within IC was 140.64 μmol, which was close to the amount of C within the end-product, CO_2_ (137.00 μmol). The value of the original amount of N within IC *vs.* the one within end-product NO_3_^−^ was 17.58 μmol/10.20 μmol. The value of the original amount of N within IC *vs.* the one within end-product NO_3_^−^ was 17.58 μmol/11.40 μmol. The above results agreed with the analysis about Figure 12, Figure 13 and Figure 14.

**Figure 16:**
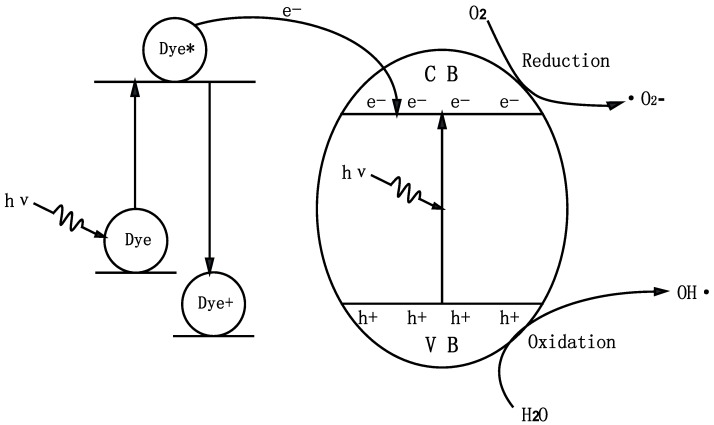
Suggested photodegradation reaction mechanism of IC with ZnBiSbO_4_ as the photocatalyst under visible light irradiation. (CB, Conduction Band; VB, Valence Band)

A mechanism scheme of the charge separation and photocatalytic reaction for ZnBiSbO_4_ is shown in Figure 16. Firstly, photoinduced holes (h^+^) and photoinduced electrons (e^−^) came into being in the surface of ZnBiSbO_4_ particles (Equation (3)). Secondly, organic pollutants (R) could be degraded into inorganic products with the effluence of h^+^ and e^−^. Many published works [56,57,58] had confirmed that two oxidative agents could be mainly concerned under visible light irradiation: OH·radicals and·O_2_^−^ radicals. Then, h^+^ reacted with R directly (Equations (4)–(6)). Besides, the effect of dye sensitization should be taken into consideration (Equations (7) and (8)); because IC could be excited by visible light, and then, the sensitizing dye molecules injected electrons into the semiconductor nanocrystallites, which were collected at a conducting surface to generate the photocurrent (Equation (9)) [59,60].

ZnBiSbO_4_ + *hν* → h^+^ + e^−^ (3)

(H_2_O ↔ H^+^ + OH^−^) + h^+^ → H^+^ +· OH (4)

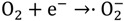
(5)


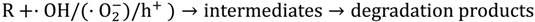
(6)

IC + *hν* → IC* (7)

IC* + ZnBiSbO_4_ → IC^+^ + ZnBiSbO_4_(e) (8)

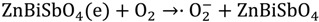
(9)

### 2.3. Phytotoxicity Properties

The phytotoxicity of the photocatalytic-treated IC wastewater was detected by examining its effect on seed germination and growth as shown in Table 4 and Table 5. We could find that when indigo carmine wastewater was straightly used for agriculture, it was so hazardous to the environment that the seeds could not survive. It could be seen from Table 4 and Table 5 that germinations, the lengths of the plumule and radicle of *Triticum aestivum* and *Sorghum vulgare* seeds went up consistently with increasing the photocatalytic reaction time. The above result revealed that the metabolites generated during the photodegradation of IC wastewater were less toxic than the compound in the original IC wastewater. Moreover, the growth conditions of the seeds cultured by IC wastewater after 220 min of photocatalytic reaction were approximate to the control group. The phytotoxicity experiment results demonstrated that the photocatalytic degradation with ZnBiSbO_4_ as a catalyst under visible light irradiation had a significant positive effect on IC wastewater treatment.

**Table 4:** The phytotoxicity study for *Sorghum vulgare* seeds by using treated IC wastewater. The results are the mean of triplicates (mean ± SD).

Parameters	*Sorghum vulgare*						
	Control	Sample of different photocatalytic reaction time (min)					
		0	40	80	120	160	220
Germination (%)	100	0	7.2 ± 0.6	21 ± 4.1	44 ± 6.5	68 ± 8.0	92 ± 3.5
Plumule (cm)	9.82 ± 1.23	0	1.15 ± 0.18	2.02 ± 0.36	4.16 ± 0.58	5.33 ± 0.79	8.54 ± 1.23
Radicle (cm)	6.79 ± 9.52	0	0.36 ± 0.12	1.04 ± 0.22	2.29 ± 0.29	3.22 ± 0.54	5.47 ± 0.89

**Table 5:** The phytotoxicity study for *Triticum aestivum* seeds by using treated IC wastewater. The results are the mean of triplicates (mean ± SD).

Parameters	*Triticum aestivum*						
	Control	Sample of different photocatalytic reaction time (min)					
		0	40	80	120	160	220
Germination (%)	100	0	5.7 ± 1.3	16 ± 2.0	37 ± 6.4	63 ± 8.7	87 ± 2.5
Plumule (cm)	8.24 ± 1.12	0	1.02 ± 0.22	1.79 ± 0.29	3.84 ± 0.68	5.11 ± 0.82	8.32 ± 1.25
Radicle (cm)	6.13 ± 1.07	0	0.31 ± 0.05	0.96 ± 0.14	2.03 ± 0.57	3.02 ± 0.44	5.25 ± 0.78

## 3. Experimental Section

### 3.1. Synthesis of Nanocatalyst

The novel photocatalysts, ZnBiSbO_4_ and CdBiYO_4_, were prepared by the solid-state reaction method. ZnO, Bi_2_O_3_ and Sb_2_O_3_ with a purity of 99.99% (Sinopharm Group Chemical Reagent Co., Ltd., Shanghai, China) were used as raw materials. In order to synthesize ZnBiSbO_4_, the precursors were stoichiometrically mixed in a quartz mortar, then pressed into small columns and put into an alumina crucible (Shenyang Crucible Co., Ltd., Shenyang, China). Finally, calcination was carried out at 800 °C for 35 h in an electric furnace (KSL 1700X, Hefei Kejing Materials Technology Co., Ltd., Hefei, China). The last step was sintering and grinding with a quartz mortar, and then, ZnBiSbO_4_ powder was fabricated. CdO, Bi_2_O_3_ and Y_2_O_3_ with a purity of 99.99% were utilized as raw materials and utilized without further purification. All powders were dried at 200 °C for 4 h before synthesis. In order to synthesize CdBiYO_4_, the precursors were stoichiometrically mixed in a quartz mortar, subsequently pressed into small columns and put into an alumina crucible. Finally, calcination was carried out at 900 °C for 36 h in an electric furnace. Nitrogen-doped titania (N-TiO_2_) catalyst with tetrabutyl titanate as a titanium precursor was prepared by using the sol-gel method at room temperature. The following procedure was that 17 mL tetrabutyl titanate and 40 mL absolute ethyl alcohol were mixed as Solution A; subsequently, Solution A was added dropwise under vigorous stirring into the Solution B that contained 40 mL absolute ethyl alcohol, 10 mL glacial acetic acid and 5 mL double distilled water to form transparent colloidal Suspension C. Subsequently, aqua ammonia with N/Ti proportion of 8 mol % was added into the resulting transparent colloidal suspension under vigorous stirring condition and stirred for 1 h. Finally, the xerogel was formed after being aged for 2 days. The xerogel was ground into powder, which was calcinated at 500 °C for 2 h; subsequently, the above powder was ground in an agate mortar and screened by a shaker to obtain N-TiO_2_ powders.

### 3.2. Characterization of ZnBiSbO_4_

The particle morphologies of ZnBiSbO_4_ were measured by transmission electron microscope (TEM, Tecnal F20 S-Twin, FEI Corporation, Hillsboro, OR, USA). The chemical composition of the compound was determined by a scanning electron microscope, which was equipped with X-ray energy dispersion spectrum (SEM-EDS, LEO 1530VP, LEO Corporation, Pegnitz, Germany) and X-ray photoelectron spectroscopy (XPS, ESCALABMK-2, VG Scientific Ltd., East Grinstead, UK). The Zn^2+^ content, Bi^3+^ content, Sb^3+^ content and O^2−^ content of ZnBiSbO_4_ and the valence state of the elements were also analyzed by X-ray photoelectron spectroscopy. The chemical composition within the depth profile of ZnBiSbO_4_ was examined by the argon ion denudation method when X-ray photoelectron spectroscopy was utilized. The particle sizes of ZnBiSbO_4_ were measured by Malvern’s mastersize-2000 particle size analyzer (Malvern Instruments Ltd., Malvern, UK). The crystalline phase of ZnBiSbO_4_ was analyzed by X-ray diffractometer (D/MAX-RB, Rigaku Corporation, Tokyo, Japan) with Cu-*K*α radiation (λ = 1.54056 Å). The patterns were collected at 295 K with a step-scan procedure in the range of 2θ = 10°–100°. The step interval was 0.02°, and the time per step was 1 s. The accelerating voltage and applied current were 40 kV and 40 mA, respectively. Fourier transform infrared spectroscopy (FTIR, Nexus, Nicolet Corporation, Madison, WI, USA) was used to examine the FTIR spectra of ZnBiSbO_4_. The UV-visible diffuse reflectance spectrum of ZnBiSbO_4_ was measured with a Shimadzu UV-2550 UV-Visible spectrometer (Shimadzu, Santa Clara, CA, USA), and BaSO_4_ was utilized as the reference material. The Brunauer–Emmett–Teller (BET) surface area was detected by nitrogen-sorption using a Micromeritics ASAP 2020 analyzer (Micromeritics, Atlanta, GA, USA).

### 3.3. Photocatalytic Activity Experiments

The photocatalytic activity of ZnBiSbO_4_ was evaluated with indigo carmine (IC) (C_16_H_8_N_2_Na_2_O_8_S_2_) (Tianjin Bodi Chemical Co., Ltd., Tianjin, China) as the model material. The photoreaction was carried out in a photochemical reaction apparatus (Nanjing Xujiang Machine Plant, Nanjing, China). The internal structure of the reaction apparatus was as following: the lamp was put into a quartz hydrazine, which was a hollow structure, and located in the middle of the reactor. The recycling water through the reactor maintained at a near constant reaction temperature (20 °C), and the solution was continuously stirred and aerated. Twelve holes were utilized to put quartz tubes evenly distributed around the lamp, and the distance between the lamp and each hole was equal. Under the condition of magnetic stirring, the photocatalyst within the IC solution was in a state of suspension. In this paper, the photocatalytic degradation of IC was performed with 0.3 g ZnBiSbO_4_ in a 300-mL, 29.3-μmol/L IC aqueous solution in quartz tubes with 500 W Xenon lamp (400 nm < λ < 800 nm) as the visible light source. Prior to visible light irradiation, the suspensions, which contained the catalyst and IC dye, were magnetically stirred in darkness for 45 min to ensure the establishment of an adsorption/desorption equilibrium among ZnBiSbO_4_, the IC dye and atmospheric oxygen. During visible light illumination, the suspension was stirred at 500 rpm, and the initial pH value of the IC solution was 7.0 without pH value adjustment in the reaction process. The above experiments were performed under oxygen-saturation conditions ([O_2_]_sat_ = 1.02 × 10^−3^ mol/L). One of the quartz tubes was taken out from the photochemical reaction apparatus at various time intervals. The suspension was filtered through 0.22-µm membrane filters. The filtrate was subsequently analyzed by a Shimadzu UV-2450 spectrometer (Shimadzu, Santa Clara, CA, USA) with a detecting wavelength at 610 nm. The experimental error was found to be within ±2.2%.

The photonic efficiency was calculated according to the following Equation (10) [61,62]:

ξ = *R*/*I*_0_ (10)

where ξ was the photonic efficiency (%), *R* was the rate of indigo carmine degradation (mol·L^−1^·s^−1^), which indicated the concentration decrement of indigo carmine within every second, and *I*_0_ was the incident photon flux (Einstein·L^−1^·s^−1^). The incident photon flux, *I*_0_, which was measured by a radiometer (Model FZ-A, Photoelectric Instrument Factory, Beijing Normal University, Beijing, China), was determined to be 4.76 × 10^−6^ Einstein·L^−1^·s^−1^ under visible light irradiation (a wavelength range of 400–700 nm).

### 3.4. Phytotoxicity Experiments

The phytotoxicity study was carried out by using *Triticum aestivum* and *Sorghum vulgare* seeds. Fifteen seeds of *Triticum aestivum* or *Sorghum vulgare* were placed over two sheets of filter paper (Whatman No. 42) in 110 × 20 mm glass petri dishes at room temperature. Subsequently, IC effluent samples (15 mL) were added to each petri dish, respectively. Control groups contained 15 mL distilled water, instead of effluents, at the same time. Germination (%) and the length of the plumule and radicle were recorded after 9 days.

## 4. Conclusions

In summary, newly synthesized photocatalyst ZnBiSbO_4_ showed higher photocatalytic activity compared with N-TiO_2_ or CdBiYO_4_ for the photocatalytic degradation of indigo carmine under visible light irradiation. The photocatalytic degradation of indigo carmine with ZnBiSbO_4_ as a catalyst followed the first-order reaction kinetics. The possible photocatalytic degradation pathway of indigo carmine was obtained. The phytotoxicity of the photocatalytic-treated IC wastewater was detected by examining its effect on seed germination and growth. The phytotoxicity experiment results demonstrated that the photocatalytic degradation with ZnBiSbO_4_ as a catalyst under visible light irradiation had a significant positive effect on IC wastewater treatment. The results obtained in our investigations proved that ZnBiSbO_4_/(visible light) photocatalysis might be regarded as a method for the practical treatment of diluted colored waste water in the environment of room-temperature and ordinary pressure.
